# Binge Drinking Intensity and Health-Related Quality of Life Among US Adult Binge Drinkers

**DOI:** 10.5888/pcd9.110204

**Published:** 2012-04-12

**Authors:** Xiao-Jun Wen, Dafna Kanny, William W. Thompson, Catherine A. Okoro, Machell Town, Lina S. Balluz

**Affiliations:** Centers for Disease Control and Prevention, Atlanta, Georgia; Division of Adult and Community Health, National Center for Chronic Disease Prevention and Health Promotion, Centers for Disease Control and Prevention; Centers for Disease Control and Prevention, Atlanta, Georgia; Centers for Disease Control and Prevention, Atlanta, Georgia; Centers for Disease Control and Prevention, Atlanta, Georgia; Centers for Disease Control and Prevention, Atlanta, Georgia

## Abstract

**Introduction:**

Binge drinking (men, ≥5 drinks, women, ≥4 on an occasion) accounts for more than half of the 79,000 annual deaths due to excessive alcohol use in the United States. The frequency of binge drinking is associated with poor health-related quality of life (HRQOL), but the association between binge drinking intensity and HRQOL is unknown. Our objective was to examine this association.

**Methods:**

We used 2008-2010 Behavioral Risk Factor Surveillance System data and multivariate linear regression models to examine the association between binge drinking intensity (largest number of drinks consumed on any occasion) among US adult binge drinkers and 2 HRQOL indicators: number of physically and mentally unhealthy days.

**Results:**

Among binge drinkers, the highest-intensity binge drinkers (women consuming ≥7 drinks and men consuming ≥8 drinks on any occasion) were more likely to report poor HRQOL than binge drinkers who reported lower levels of intensity (women who consumed 4 drinks and men who consumed 5 drinks on any occasion). On average, female binge drinkers reported more physically and mentally unhealthy days (2.8 d and 5.1 d, respectively) than male binge drinkers (2.5 d and 3.6 d, respectively). After adjustment for confounding factors, women who consumed ≥7 drinks on any occasion reported more mentally unhealthy days (6.3 d) than women who consumed 4 drinks (4.6 d). Compared with male binge drinkers across the age groups, female binge drinkers had a significantly higher mean number of mentally unhealthy days.

**Conclusion:**

Our findings underscore the importance of implementing effective population-level strategies to prevent binge drinking and improve HRQOL.

## MEDSCAPE CME

Medscape, LLC is pleased to provide online continuing medical education (CME) for this journal article, allowing clinicians the opportunity to earn CME credit.

This activity has been planned and implemented in accordance with the Essential Areas and policies of the Accreditation Council for Continuing Medical Education through the joint sponsorship of Medscape, LLC and Preventing Chronic Disease. Medscape, LLC is accredited by the ACCME to provide continuing medical education for physicians. 

Medscape, LLC designates this Journal-based CME activity for a maximum of 1 **AMA PRA Category 1 Credit(s)™**. Physicians should claim only the credit commensurate with the extent of their participation in the activity.

All other clinicians completing this activity will be issued a certificate of participation. To participate in this journal CME activity: (1) review the learning objectives and author disclosures; (2) study the education content; (3) take the post-test with a 70% minimum passing score and complete the evaluation at www.medscape.org/journal/pcd (4) view/print certificate.

**Release date: April 11, 2012; Expiration date: April 11, 2013**

### Learning Objectives

Upon completion of this activity, participants will be able to:

- Describe the association between binge drinking intensity among US adult binge drinkers and HRQOL, based on a cross-sectional US study
- Compare male and female binge drinkers in terms of the number of physically and mentally unhealthy days, based on a cross-sectional US study
- Describe factors affecting HRQOL in female binge drinkers, based on a cross-sectional US study

**CME EDITOR**

Ellen Taratus, Editor, *Preventing Chronic Disease*. Disclosure: Ellen Taratus has disclosed no relevant financial relationships.

**CME AUTHOR**

Laurie Barclay, MD. Freelance writer and reviewer, Medscape, LLC. Disclosure: Laurie Barclay, MD, has disclosed no relevant financial relationships. 

**AUTHORS AND CREDENTIALS**

Disclosures: Xiao-Jun Wen, MD; Dafna Kanny, PhD; William W. Thompson, PhD; Catherine A. Okoro, MS; Machell Town, MS; and Lina S. Balluz, ScD, MPH have disclosed no relevant financial relationships.

Affiliations: Dafna Kanny, Xiao-Jun Wen, William W. Thompson, Catherine A. Okoro, Machell Town, Lina S. Balluz, Centers for Disease Control and Prevention, Atlanta, Georgia.

## Introduction

Excessive alcohol consumption, including binge and underage drinking, is the third leading preventable cause of death in the United States, and binge drinking, defined for women as consuming 4 or more alcoholic drinks on an occasion and for men as consuming 5 or more drinks on an occasion, accounts for more than half of the 79,000 annual deaths due to excessive drinking (1,2). Binge drinking is a common form of excessive alcohol use in the United States (1). According to 2009 Behavioral Risk Factor Surveillance System (BRFSS) data, an estimated 15.2% of adults (20.7% of men and 10.0% of women) are binge drinkers (2). Among some population groups, such as people aged 18 to 34 years, the prevalence of binge drinking is even higher (2,3). The frequency (4) (ie, number of binge drinking episodes within a defined time period) and intensity (4) (ie, number of drinks consumed per episode) of binge drinking are 2 measures used to examine the adverse health effects for this risk behavior (5). Several studies have demonstrated that risk of alcohol-related illness and death increases with the intensity of binge drinking (6-8).

Several studies have examined the association between health-related quality of life (HRQOL) and alcohol use (9,10) and the association between binge drinking and certain risk behaviors (eg, alcohol-impaired driving and violence) (11,12). In 2004, 1 study (13) reported that frequent binge drinking was associated with significantly worse HRQOL and mental distress, including stress, depression, and emotional problems. However, the relationship between the intensity of binge drinking per episode and HRQOL has not been examined. The primary objective of this study was to examine the association between HRQOL and the intensity of binge drinking among US adult binge drinkers. A secondary objective was to compare sex differences in HRQOL by sociodemographic characteristics and the intensity of binge drinking.

## Methods

We used 2008-2010 BRFSS data and multivariate linear regression models to assess the relationship between binge drinking intensity and HRQOL.

### Data source

The BRFSS survey is a state-based, continuous random-digit–dialed telephone survey that collects information on risk behaviors and health conditions from noninstitutionalized adults aged 18 or older in 50 states; Washington, DC; and US territories. Trained interviewers collect data monthly by using an independent probability sample of households with landline telephones. The characteristics, survey design, and random sampling of BRFSS are described elsewhere (14). The validity and reliability of BRFSS data have been demonstrated (15,16).

### Assessment of binge drinking

We defined binge drinking by using the question, "Considering all types of alcoholic beverages, how many times during the past 30 days did you have [5 for men, 4 for women] or more drinks on an occasion?" We assessed the intensity of binge drinking among binge drinkers by using the question, "During the past 30 days, what is the largest number of drinks you had on any occasion?" We calculated the median largest number of drinks consumed and then categorized female binge drinkers into 4 groups (4, 5, 6, and 7 or more drinks on any occasion during the past 30 days); and male binge drinkers into 4 groups (5, 6, 7, and 8 or more drinks on any occasion during the past 30 days).

### Assessment and definition of HRQOL

We analyzed 2 of the HRQOL questions that are administered annually in the core BRFSS survey: 1) "Now thinking about your physical health, which includes physical illness and injury, for how many days during the past 30 days was your physical health not good?" and 2) "Now thinking about your mental health, which includes stress, depression, and problems with emotions, for how many days during the past 30 days was your mental health not good?" The reliability and validity of these measures have been described (17). We calculated physically and mentally unhealthy days according to the methodology and computer program code published by the Centers for Disease Control and Prevention (18).

### Participants

We examined 2008-2010 BRFSS data from respondents who resided in any of the 50 states and Washington, DC, and reported at least 1 binge drinking episode in the past 30 days. Of the 133,353 binge drinkers, 76,269 (66.6%) were men. The median response rates (calculated according to Council of American Survey Research Organizations methods) were 53.3% (range, 35.8%-65.9%) for 2008, 52.5% (range, 37.9%-68.9%) for 2009, and 54.6% (range, 39.1%-68.8%) for 2010. The cooperation rates were 75.0% (range, 59.3%-87.8%) for 2008, 75.0% (range, 55.0%-88.0%) for 2009, and 76.9% (range, 56.8%-86.1%) for 2010.

### Sociodemographic characteristics

We analyzed binge drinking intensity and HRQOL by the following sociodemographic variables: age group (18-44 y and ≥45 y); race/ethnicity (non-Hispanic white, non-Hispanic black, Hispanic, and non-Hispanic other); marital status (married, previously married, and never married); education (<high school diploma, high school diploma, some college or technical school, and ≥college degree); employment (employed, unemployed, homemaker/student, retired, and unable to work); and income (<$25,000, $25,000 to <$50,000, and ≥$50,000).

### Data analysis

We calculated the mean number of physically and mentally unhealthy days by sociodemographic characteristic and by binge drinking intensity. We conducted multivariate linear regression analyses for the predicted mean number of unhealthy days by sex and largest number of drinks consumed while adjusting for age, race/ethnicity, education, marital status, income, and employment. Because of the complex sampling design of BRFSS, we used SUDAAN version 9.2 (Research Triangle Institute, Research Triangle Park, North Carolina) to calculate weighted prevalence estimates and 95% confidence intervals (CIs). We used nonoverlapping 95% CIs as the criteria for statistical significance.

## Results

The median largest number of drinks consumed was 5 (range, 4-14 drinks) for female binge drinkers and 6 (range, 5-29 drinks) for male binge drinkers. Among women, the median intensity of binge drinking was similar among sociodemographic groups. However, among men, the median intensity of binge drinking was higher among men aged 18 to 44 (7 drinks) than among men aged 45 or older (6 drinks), among men who never married (8 drinks) than among men who were married (6 drinks), and among men who were homemaker/students (8 drinks) than among men who were retired or employed (6 drinks).

Overall, female binge drinkers reported more unhealthy days (2.8 physically unhealthy days and 5.1 mentally unhealthy days) than male binge drinkers (2.5 physically unhealthy days and 3.6 mentally unhealthy days) (Table 1). The mean number of mentally unhealthy days among women and men aged 18 to 44 was significantly higher than the mean number for those aged 45 or older, whereas the mean number of physically unhealthy days among men and women aged 45 or older was significantly higher than the mean number for the younger age group. Men and women who were previously married had a significantly higher mean number of physically and mentally unhealthy days than men and women who were married or never married.

Female binge drinkers who consumed 7 or more drinks on any occasion reported significantly more unhealthy days (3.2 physical and 6.9 mental) compared with those who were binge drinking at a lower intensity (Table 1). Similarly, male binge drinkers who consumed 8 or more drinks on any occasion reported significantly more mentally unhealthy days (4.3 d) compared with those who were binge drinking at a lower intensity.

After adjustment for potential confounding factors (age, race/ethnicity, education, marital status, income, and employment), female binge drinkers who consumed 7 or more drinks had more mentally unhealthy days compared with female binge drinkers who consumed 4 drinks, and male binge drinkers who consumed 8 or more drinks had more mentally unhealthy days compared with male binge drinkers who consumed 5 drinks (Table 2).

**Figure 1.**
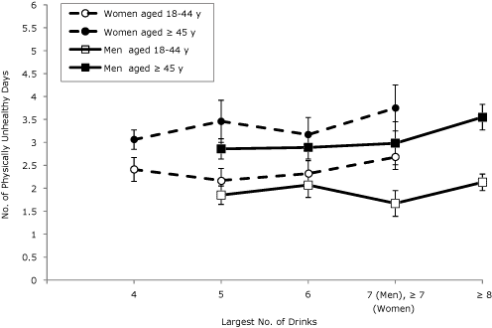
Predicted mean number of physically unhealthy days by sex and age among binge drinkers, adjusted for race/ethnicity, education, marital status, income, employment, and survey year. Binge drinking is defined as 4 or more drinks for women and 5 drinks or more drinks for men on an occasion. Error bars indicate 95% confidence intervals. Data are from 50 states and Washington, DC; Behavioral Risk Factor Surveillance System, 2008-2010.

**Figure 2.**
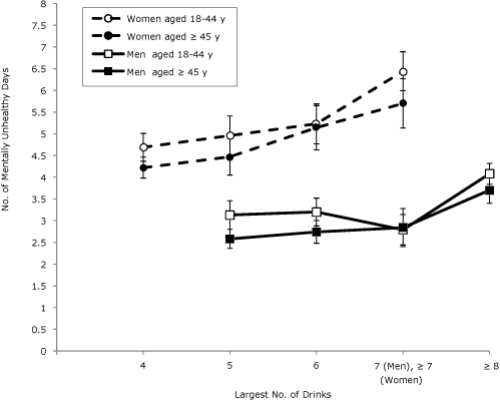
Predicted mean number of mentally unhealthy days by sex and age among binge drinkers, adjusted for race/ethnicity, education, marital status, income, employment, and survey year. Binge drinking is defined as 4 or more drinks for women and 5 drinks or more drinks for men on an occasion. Error bars indicate 95% confidence intervals. Data are from 50 states and Washington, DC; Behavioral Risk Factor Surveillance System, 2008-2010.

In general, among all age groups for both sexes, the mean number of physically unhealthy days was associated with binge drinking intensity (Figure 1). The mean number of physically unhealthy days among both sexes aged 45 older was higher than for those aged 18 to 44. In general, among all age groups for both sexes, the mean number of mentally unhealthy days was associated with binge drinking intensity (Figure 2). The mean number of mentally unhealthy days among female binge drinkers was significantly higher than the mean number for male binge drinkers. We found no significant interactions between age and intensity of binge drinking in any of the linear regression models.

**Table:** 

Characteristic	Predicted Mean Physically Unhealthy Days				

	4 drinks	5 drinks	6 drinks	7 drinks (men), ≥7 drinks (women)	≥8 drinks
Women aged 18-44 y	2.41 (2.15-2.67)	2.17 (1.91-2.44)	2.32 (2.04-2.60)	2.68 (2.41-2.94)	NA
Women aged ≥45 y	3.06 (2.85-3.27)	3.46 (3.00-3.92)	3.17 (2.80-3.55)	3.75 (3.25-4.25)	NA
Men aged 18-44 y	NA	1.85 (1.65-2.06)	2.07 (1.80-2.35)	1.67 (1.39-1.96)	2.13 (1.95-2.30)
Men aged ≥45 y	NA	2.86 (2.64-3.07)	2.89 (2.64-3.14)	2.98 (2.51-3.45)	3.55 (3.27-3.84)

**Table:** 

Characteristic	Predicted Mean Mentally Unhealthy Days				

	4 drinks	5 drinks	6 drinks	7 drinks (men), ≥7 drinks (women)	≥8 drinks
Women aged 18-44 y	4.69 (4.37-5.01)	4.96 (4.51-5.41)	5.23 (4.77-5.68)	6.44 (5.99-6.89)	NA
Women aged ≥45 y	4.22 (3.98-4.46)	4.47 (4.05-4.89)	5.14 (4.63-5.66)	5.70 (5.13-6.28)	NA
Men aged 18-44 y	NA	3.13 (2.80-3.46)	3.20 (2.88-3.52)	2.79 (2.44-3.14)	4.08 (3.84-4.31)
Men aged ≥45 y	NA	2.58 (2.36-2.80)	2.74 (2.48-3.00)	2.84 (2.40-3.29)	3.70 (3.40-4.00)

## Discussion

Although several studies have examined either sex-specific or alcohol-specific effects for HRQOL (13,19-21), this is the first study to examine the association between binge drinking intensity and HRQOL by sex. Adults who had high-intensity levels of binge drinking were more likely to report poor HRQOL than adults who had lower-intensity levels of binge drinking. This pattern was found for 2 measures of HRQOL — physically and mentally unhealthy days.

Among female binge drinkers, the highest-intensity binge drinkers had 37% more mentally unhealthy days than the lowest-intensity binge drinkers. This estimate of 1 or 2 additional unhealthy days per month is considered a meaningful difference in HRQOL (22).

We also found age effects for physically unhealthy days for both sexes; those aged 45 or older had significantly more physically unhealthy days than those aged 18 to 44. Age effects are likely due in part to the development of chronic conditions that increasingly affect health and well-being across the life span (23,24). The frequency and intensity of alcohol consumption are both important indicators for measuring and assessing the effect of binge drinking (5). A previous study found that poor HRQOL was associated with frequent binge drinking (13). Our study demonstrated that poor HRQOL (physically and mentally unhealthy days) was associated with the intensity of binge drinking among adults who reported binge drinking.

This study has several limitations. First, BRFSS is a landline telephone survey; therefore, people with cellular telephones only or no landline telephones are excluded, which may result in sampling bias. Studies show that an increasing proportion of young adults aged 18 to 34 use cellular telephones exclusively (25) and that the prevalence of binge drinking is approximately one-third higher among cellular telephone users than landline respondents to the BRFSS (2). Second, BRFSS data are self-reported and may be subject to recall and social desirability biases (26). A recent study based on state alcohol sales found that BRFSS identifies only 22% to 32% of presumed alcohol consumption (27). Third, this study is cross-sectional; therefore, any cause and effect between poor HRQOL and level of binge drinking intensity cannot be inferred. Fourth, a previous study (13) demonstrated that the frequency of binge drinking was associated with HRQOL, whereas our study investigated the relationship between the intensity of binge drinking and HRQOL. Further studies are needed to explore the effects of both frequency and intensity of binge drinking on HRQOL to better understand sex-specific effects.

This study also has several strengths. First, to our knowledge, it is the first study to assess the relationship between intensity of binge drinking and physical and mental health components of HRQOL. Second, the large sample of binge drinkers and the BRFSS sampling design suggest that these associations would be similar to those for all noninstitutionalized adult binge drinkers in the United States.

The results of this study support the recommendations of the US Preventive Services Task Force (28) to implement screening and counseling for alcohol misuse, including binge drinking, among adults by physicians and other health care providers. Such screening and counseling can highlight the negative consequences of binge drinking on current physical and mental health. In addition, these results support the use of binge drinking intensity as a measure that could be monitored when implementing and evaluating evidence-based population-level intervention strategies, such as increasing alcohol excise taxes (29) and limiting the density of alcohol sales outlets (30) to reduce drinking intensity and improve HRQOL.

## Figures and Tables

**Table:** 

1. The activity supported the learning objectives.				
Strongly Disagree				Strongly Agree
1	2	3	4	5
2. The material was organized clearly for learning to occur.				
Strongly Disagree				Strongly Agree
1	2	3	4	5
3. The content learned from this activity will impact my practice.				
Strongly Disagree				Strongly Agree
1	2	3	4	5
4. The activity was presented objectively and free of commercial bias.				
Strongly Disagree				Strongly Agree
1	2	3	4	5

**Table 1.** Mean Unhealthy Days by Selected Characteristics and Binge Drinking Intensity Among US Adult Binge Drinkers, 2008-2010^a^

Characteristic	N^b^	Physically Unhealthy Days Mean (95% CI)	Mentally Unhealthy Days Mean (95% CI)
**Women**			
**Overall**	56,656	2.8 (2.7-2.9)	5.1 (5.0-5.3)
**Age, y**			
18-44	27,262	2.5 (2.4-2.6)	5.4 (5.2-5.6)
≥45	29,394	3.4 (3.2-3.5)	4.5 (4.4-4.7)
**Race/ethnicity**			
White non-Hispanic	46,539	2.6 (2.5-2.7)	4.8 (4.7-4.9)
Black non-Hispanic	3,660	3.5 (2.9-4.1)	7.0 (6.3-7.7)
Hispanic	3,173	2.9 (2.5-3.3)	5.6 (5.0-6.3)
Other non-Hispanic	2,995	3.7 (3.2-4.2)	6.2 (5.5-6.9)
**Marital status**			
Married	32,766	2.4 (2.3-2.5)	4.3 (4.2-4.5)
Previously married	14,074	4.3 (4.1-4.6)	7.1 (6.8-7.4)
Never married	9,723	2.8 (2.5-3.0)	5.7 (5.4-6.1)
**Education**			
<high school diploma	3,031	5.1 (4.6-5.7)	8.3 (7.5-9.0)
High school diploma	14,937	3.5 (3.3-3.7)	6.3 (5.9-6.6)
Some college or technical school	17,085	2.8 (2.6-3.0)	5.4 (5.2-5.7)
≥College degree	21,571	1.9 (1.8-2.0)	3.7 (3.5-3.9)
**Employment**			
Employed	38,873	2.1 (2.0-2.2)	4.6 (4.4-4.7)
Unemployed	3,960	4.4 (3.9-4.8)	8.6 (8.0-9.1)
Homemaker/student	6,756	2.7 (2.4-3.0)	5.2 (4.9-5.6)
Retired	5,140	3.5 (3.2-3.9)	2.9 (2.6-3.3)
Unable to work	1,851	14.0 (12.9-15.2)	13.4 (12.3-14.5)
**Income, $**			
<25,000	11,178	4.9 (4.6-5.2)	8.1 (7.7-8.5)
25,000 to <50,000	13,116	2.9 (2.7-3.0)	5.8 (5.5-6.1)
≥50,000	27,977	1.9 (1.8-2.1)	3.7 (3.6-3.9)
**Largest no. of drinks consumed on any occasion**			
4	22,779	2.5 (2.3-2.7)	4.3 (4.1-4.5)
5	10,413	2.5 (2.3-2.7)	4.8 (4.4-5.1)
6	8,692	2.7 (2.4-2.9)	5.4 (5.1-5.8)
≥7	8,559	3.2 (3.0-3.4)	6.9 (6.5-7.2)
**Men**			
**Overall**	75,564	2.5 (2.4-2.5)	3.6 (3.5-3.7)
**Age, y**			
18-44	33,850	2.1 (2.0-2.2)	3.8 (3.6-3.9)
≥45	41,714	3.2 (3.1-3.4)	3.1 (3.0-3.2)
**Race/ethnicity**			
White non-Hispanic	62,007	2.3 (2.2-2.4)	3.3 (3.2-3.5)
Black non-Hispanic	3,513	3.0 (2.6-3.3)	4.8 (4.2-5.4)
Hispanic	5,115	2.7 (2.4-2.9)	3.8 (3.4-4.1)
Other non-Hispanic	4,290	3.0 (2.6-3.4)	4.1 (3.6-4.6)
**Marital status**			
Married	46,761	2.1 (2.0-2.2)	2.9 (2.8-3.1)
Previously married	14,549	4.4 (4.1-4.8)	5.4 (5.1-5.8)
Never married	14,115	2.4 (2.2-2.5)	4.2 (4.0-4.5)
**Education**			
<high school diploma	5,610	4.2 (3.8-4.6)	5.4 (4.9-5.9)
High school diploma	23,512	2.9 (2.7-3.1)	4.0 (3.8-4.3)
Some college	20,231	2.4 (2.2-2.5)	3.7 (3.5-3.9)
≥College degree	26,147	1.6 (1.5-1.7)	2.5 (2.3-2.6)
**Employment**			
Employed	55,499	1.8 (1.7-1.9)	3.0 (2.9-3.1)
Unemployed	5,924	3.8 (3.4-4.2)	6.4 (6.0-6.9)
Homemaker/student	1,780	2.0 (1.7-2.3)	3.7 (3.2-4.2)
Retired	9,603	3.7 (3.4-4.0)	2.3 (2.1-2.6)
Unable to work	2,632	14.5 (13.4-15.7)	11.2 (10.2-12.3)
**Income, $**			
<25,000	12,776	4.6 (4.3-4.9)	5.8 (5.4-6.1)
25,000 to <50,000	17,884	2.5 (2.3-2.7)	3.7 (3.5-3.9)
≥50,000	40,249	1.7 (1.6-1.7)	2.6 (2.5-2.8)
**Largest no. of drinks consumed on any occasion**			
5	18,863	2.2 (2.0-2.3)	2.8 (2.6-3.0)
6	16,292	2.3 (2.1-2.5)	2.9 (2.7-3.1)
7	5,454	2.0 (1.7-2.2)	2.8 (2.5-3.1)
≥8	26,857	2.6 (2.5-2.8)	4.3 (4.1-4.5)

Abbreviation: CI, confidence interval.

a Data from 50 states and Washington, DC, 2008-2010 Behavioral Risk Factor Surveillance System. Binge drinking defined as 4 or more drinks for women and 5 drinks or more drinks for men on an occasion.

b Sample sizes vary because of missing values in mentally unhealthy days and physically unhealthy days.

**Table 2.** Association Between Number of Unhealthy Days and Binge Drinking Intensity by Sex Among Binge Drinkers, 2008-2010^a^

Largest No. of Drinks Consumed on Any Occasion	Physically Unhealthy Days		Mentally Unhealthy Days	

	Predicted Mean (95% CI)	β (*P* Value)	Predicted Mean (95% CI)	β (*P* Value)
**Women**				
4	2.6 (2.3-2.8)	1 [Reference]	4.6 (4.3-4.8)	1 [Reference]
5	2.6 (2.3-2.8)	−0.01 (.97)	4.8 (4.4-5.2)	0.24 (.22)
6	2.6 (2.3-2.9)	0.02 (.89)	5.2 (4.8-5.6)	0.61 (.004)
≥7	3.0 (2.7-3.3)	0.41 (.009)	6.3 (5.8-6.7)	1.69 (.001)
**Men**				
5	2.1 (1.9-2.3)	1 [Reference]	2.9 (2.7-3.2)	1 [Reference]
6	2.3 (2.1-2.6)	0.19 (.10)	3.1 (2.8-3.3)	0.11 (.44)
7	2.1 (1.8-2.4)	−0.04 (.78)	2.8 (2.5-3.1)	−0.16 (.36)
≥8	2.6 (2.4-2.7)	0.42 (<.001)	4.0 (3.7-4.2)	1.01 (<.001)

Abbreviation: CI, confidence interval.

a Data from 50 states and Washington, DC, 2008-2010 Behavioral Risk Factor Surveillance System. Binge drinking defined as 4 or more drinks for women and 5 drinks or more drinks for men on an occasion. Sample size is 46,764 female binge drinkers and 63,223 male binge drinkers. Model adjusted for age, race/ethnicity, education, marital status, income, and employment.

## Post-Test Information

To obtain credit, you should first read the journal article. After reading the article, you should be able to answer the following, related, multiple-choice questions. To complete the questions (with a minimum 70% passing score) and earn continuing medical education (CME) credit, please go to http://www.medscape.org/journal/pcd. Credit cannot be obtained for tests completed on paper, although you may use the worksheet below to keep a record of your answers. You must be a registered user on Medscape.org. If you are not registered on Medscape.org, please click on the "Register" link on the right hand side of the website to register. Only one answer is correct for each question. Once you successfully answer all post-test questions you will be able to view and/or print your certificate. For questions regarding the content of this activity, contact the accredited provider, CME@medscape.net. For technical assistance, contact CME@webmd.net. American Medical Association's Physician's Recognition Award (AMA PRA) credits are accepted in the US as evidence of participation in CME activities. For further information on this award, please refer to http://www.ama-assn.org/ama/pub/category/2922.html. The AMA has determined that physicians not licensed in the US who participate in this CME activity are eligible for AMA PRA Category 1 Credits™. Through agreements that the AMA has made with agencies in some countries, AMA PRA credit may be acceptable as evidence of participation in CME activities. If you are not licensed in the US, please complete the questions online, print the AMA PRA CME credit certificate and present it to your national medical association for review.

## Post-Test Questions

### Article Title: Binge Drinking Intensity Linked to Health-Related Quality of Life

**CME Questions**

1. Based on the study by Dr. Wen and colleagues, which of the following statements about the association between binge drinking intensity among US adult binge drinkers and health-related quality of life (HRQOL) is **most likely** correct?
  - Highest-intensity binge drinkers were defined as women consuming ≥ 4 drinks and men consuming ≥ 5 drinks on any occasion
  - Frequency rather than severity of binge drinking was a better predictor of HRQOL
  - Intensity of binge drinking was a predictor of HRQOL among men but not among women
  - Highest-intensity binge drinkers were more likely to report poor HRQOL than binge drinkers who reported lower levels of intensity
2. You are a consultant to an alcohol abuse intervention program. Based on the study by Dr. Wen and colleagues, which of the following statements about the number of physically and mentally unhealthy days per month linked to binge drinking is **most likely** to appear in your report?
  - Female binge drinkers reported fewer physically unhealthy days than did male binge drinkers
  - Female binge drinkers reported 5.1 mentally unhealthy days
  - Female binge drinkers reported fewer mentally unhealthy days than did male binge drinkers
  - Male binge drinkers reported more physically unhealthy days than mentally unhealthy days
3. Based on the study by Dr. Wen and colleagues, which of the following statements about factors affecting HRQOL in female binge drinkers would be **most likely** to appear in your report?
  - After adjustment for confounding factors, women who consumed ≥ 7 drinks on any occasion did not report more mentally unhealthy days than women who consumed 4 drinks
  - Women who consumed ≥ 7 drinks on any occasion reported more than 6 mentally unhealthy days per month
  - Women who consumed 4 drinks on any occasion reported 2.5 mentally unhealthy days per month
  - Preventing binge drinking in women is unlikely to improve HRQOL.

**Evaluation**
